# Ribosome-Engineered *Lacticaseibacillus rhamnosus* Strain GG Exhibits Cell Surface Glyceraldehyde-3-Phosphate Dehydrogenase Accumulation and Enhanced Adhesion to Human Colonic Mucin

**DOI:** 10.1128/AEM.01448-20

**Published:** 2020-10-01

**Authors:** Minori Ishida, Fu Namai, Suguru Shigemori, Shoko Kajikawa, Masami Tsukagoshi, Takashi Sato, Tasuku Ogita, Takeshi Shimosato

**Affiliations:** a Department of Biomolecular Innovation, Institute for Biomedical Sciences, Shinshu University, Kamiina, Nagano, Japan; University of Helsinki

**Keywords:** GAPDH, human colonic mucin, *Lacticaseibacillus rhamnosus* GG, ribosome engineering

## Abstract

Differences in individual host responses have emerged as an issue regarding the health benefits of probiotics. Here, we applied ribosome engineering (RE) technology, developed in an actinomycete study, to *Lacticaseibacillus rhamnosus* GG (LGG). RE can effectively enhance microbial potential by using antibiotics to induce spontaneous mutations in the ribosome and/or RNA polymerase. In this study, we identified eight types of streptomycin resistance mutations in the LGG *rpsL* gene, which encodes ribosomal protein S12. Notably, LGG harboring the K56N mutant (LGG-MT_K56N_) expressed high levels of glyceraldehyde-3-phosphate dehydrogenase (GAPDH) on the cell surface compared with the LGG wild type (LGG-WT). GAPDH plays a key role in colonic mucin adhesion. Indeed, LGG-MT_K56N_ significantly increased type A human colonic mucin adhesion compared to LGG-WT in experiments using the Biacore system. The ability to adhere to the colon is an important property of probiotics; thus, these results suggest that RE is an effective breeding strategy for probiotic lactic acid bacteria.

**IMPORTANCE** We sought to apply ribosome engineering (RE) to probiotic lactic acid bacteria and to verify RE’s impact. Here, we showed that one mutant of RE *Lacticaseibacillus rhamnosus* GG (LGG-MT_K56N_) bore a GAPDH on the cell surface; the GAPDH was exported via an ABC transporter. Compared to the wild-type parent, LGG-MT_K56N_ adhered more strongly to human colonic mucin and exhibited a distinct cell size and shape. These findings demonstrate that RE in LGG-MT_K56N_ yielded dramatic changes in protein synthesis, protein transport, and cell morphology and affected adherence to human colonic mucin.

## Abstract (precis)

We sought to apply ribosome engineering (RE) to probiotic lactic acid bacteria and to verify RE’s impact. Here, we showed that one mutant of RE *Lacticaseibacillus rhamnosus* GG (LGG-MT_K56N_) bore a GAPDH on the cell surface; the GAPDH was exported via an ABC transporter. Compared to the wild-type parent, LGG-MT_K56N_ adhered more strongly to human colonic mucin and exhibited a distinct cell size and shape. These findings demonstrate that RE in LGG-MT_K56N_ yielded dramatic changes in protein synthesis, protein transport, and cell morphology and affected adherence to human colonic mucin.

## INTRODUCTION

Lactic acid bacteria (LAB) have been human commensals since ancient times. In light of their long history of use in food processing, certain lactobacillus strains have been classified by the Food and Drug Administration (FDA) as generally recognized as safe (GRAS) (1). The functionality of LAB, such as immunoregulatory and metabolic promoting effects, has been studied worldwide (2, 3). Kalliomaki et al. reported that Lactobacillus rhamnosus, or Lacticaseibacillus rhamnosus GG (LGG) according to the recent *Lactobacillus* taxonomy change (4), intake during pregnancy resulted in a lower incidence of atopic dermatitis in infants than placebo (5). However, a 2006 study by Fölster-Holst et al. identified no significant differences in severity scoring of atopic dermatitis (SCORAD) when comparing infants who consumed LGG to those who consumed a placebo (6). In addition, the administration of LGG in adults with hay fever or food allergy did not demonstrate efficacy in the palliation of symptoms (7). The populations in these studies, which showed different outcomes, varied in age (including pregnant women, young children, and adults) and ethnicity. Thus, it has been pointed out that even effective probiotic strains show variability in efficacy depending on the administration method (administration timing and dose) and host background (ethnicity and living conditions) (8, 9).

To improve the quality of life (QOL) of humans, exploratory studies of robust functional LAB are increasingly favored. In this context, screening tests have been widely conducted to identify new and useful probiotic strains; however, this is a time-consuming and laborious process. On the other hand, microbial breeding technology has been concurrently developed, permitting the production of bacterial mutants by various methods such as gene editing and engineering, UV irradiation (10), and chemical mutagenesis (11–13). However, gene editing and engineering are controversial in food-grade applications, while some other technologies are known to pose risks to handlers. Therefore, we employed ribosome engineering (RE) as a simpler and safer breeding strategy for probiotic LAB. RE has been studied in actinomycetes, Bacillus subtilis, and Escherichia coli and has been shown to be an effective technique for enhancing microbial potential (14–16). This method targets ribosomal proteins and/or RNA polymerase and has been used to enhance the production of secondary metabolites by microorganisms (17). Antibiotic-resistant variants have been reported to harbor mutations in specific genes, depending on the selective antibiotic used; notably, streptomycin often selects for strains harboring mutations in the *rpsL* gene, which encodes ribosomal protein S12 (18). Although RE is a simple and effective microbial breeding strategy, to the best of our knowledge there have been no reports of its application to LAB. The purpose of this study was to investigate RE as a breeding strategy for probiotic LAB, by applying RE to LGG and evaluating the characteristics of the resultant mutants.

## RESULTS

### Isolation of LGG-MTs.

Individual colonies were identified on medium containing 256 μg/ml streptomycin, suggesting that the mutants possessed streptomycin MICs that exceed the 256-μg/ml MIC of the parent (referred to as wild-type LGG [LGG-WT]). The colonies (145 in total) that retained the ability to grow on de Man, Rogosa, and Sharpe (MRS) agar containing streptomycin (256 μg/ml) upon restreaking were assigned numbers (see Table S1 in the supplemental material) and then reinoculated on MRS agar containing streptomycin (256 μg/ml). Satellite colonies and streptomycin-requiring strains were excluded, and the remaining isolates (designated LGG-MTs; 132 in total) were collected. To characterize the LGG-MTs, the *rpsL* gene (encoding ribosomal protein S12) was amplified by direct colony PCR and then subjected to DNA sequencing. This analysis confirmed the presence of *rpsL* mutations in 125 of 132 LGG-MTs; a total of 10 distinct nucleotide mutations were detected. At the protein level, these base substitutions corresponded to 8 distinct amino acid substitutions, such that (in total) 124 of 132 LGG-MTs could be demonstrated to encode mutant S12 proteins with specific amino acid changes (Table 1). Seven of the 8 amino acid substitutions corresponded to replacement of Lys residues, either Lys56 (4 of 8) or Lys101 (3 of 8). The remaining amino acid substitution corresponded to an Arg-to-Cys replacement at residue 99. The frequency of each mutant is shown in Table 1. In particular, LGG-MT_K56N_ was the most abundant, accounting for 46.0% of the colonies isolated. The 4 LGG-MTs harboring mutations at *rpsL* codon 56 (LGG-MT_K56N_, LGG-MT_K56T_, LGG-MT_K56M_, and LGG-MT_K56R_) were able to grow at streptomycin concentrations as high as 65,536 μg/ml. The three LGG-MTs harboring mutations at *rpsL* codon 101 (LGG-MT_K101E_, LGG-MT_K101R_, and LGG-MT_K101M_) exhibited streptomycin MICs of 2,048 to 4,096 μg/ml. The LGG-MT harboring a mutation at *rpsL* codon 99 (LGG-MT_R99C_) had a streptomycin MIC of 512 μg/ml (Table 1).

**TABLE 1:** Profiles of LGG-MTs isolated from RE for streptomycin resistance

Colony no.	Relevant genotype	Frequency of mutants^*a*^	MIC (μg/ml)
Wild type (WT)			256
001	*rpsL*(K56N)	57	>65,536
006	*rpsL*(K101E)	35	4,096
010	*rpsL*(K56T)	8	>65,536
022	*rpsL*(K101R)	3	2,048
027	*rpsL*(K56M)	5	>65,536
045	*rpsL*(K56R)	14	>65,536
112	*rpsL*(K101M)	1	4,096
134	*rpsL*(R99C)	1	512

a Number of events among the 124 confirmed LGG-MTs that were isolated.

### Identification of secreted protein with a mass of approximately 40 kDa.

**FIG 1:**
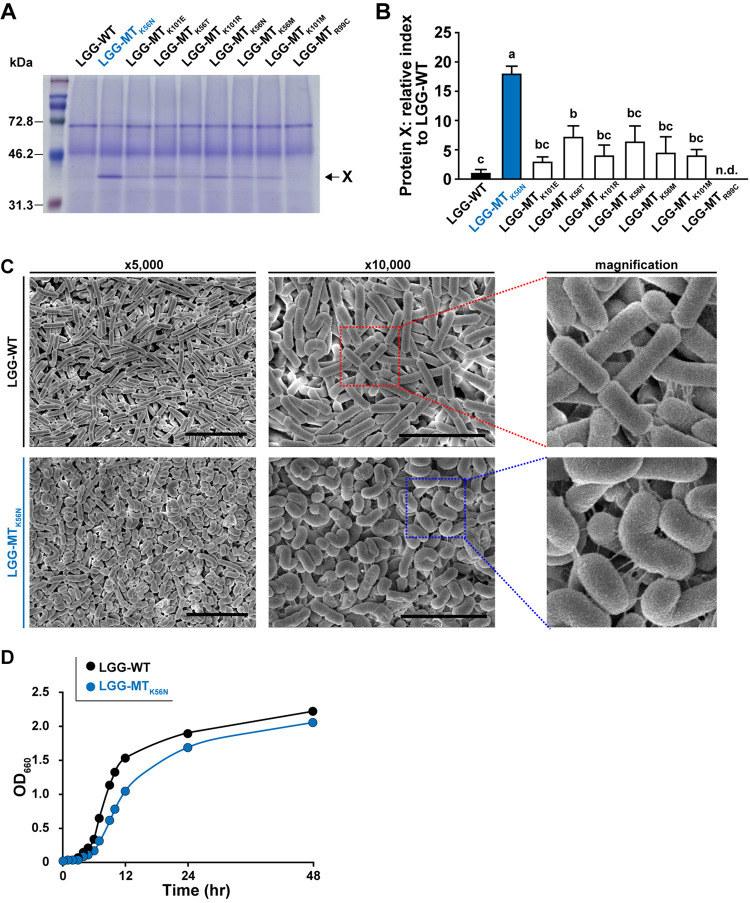
(A) The secreted proteins present in culture supernatants (spent medium) were analyzed by SDS-PAGE with CBB staining. The arrow indicates the 40-kDa protein (X) that appears to accumulate in the LGG-MTs but not in the parent. A representative image from one of three independent experiments is shown. (B) Densitometric analysis of gels from three independent SDS-PAGE experiments was used to quantitate protein X production; protein levels are normalized to that in the LGG-WT parent. Letters (i.e., a, b, and c) represent significant differences (*P < *0.05) by a two-tailed one-way ANOVA. Data are means and SD (*n* = 3). n.d., not detected. (C) SEM photographs of LGG-WT and LGG-MT_K56N_ cells. Images on the right are greater magnifications of the indicated areas in the middle panels. Bar = 5 μm. (D) Growth curves of LGG-WT and LGG-MT_K56N_.

LGG-WT and LGG-MTs were cultured in MRS without streptomycin for 24 h. After incubation, the spent medium and bacteria from individual cultures were separated; each was subjected to protein precipitation to facilitate characterization of secreted proteins and cellular proteins, respectively. The resulting protein preparations were analyzed by SDS-PAGE and Coomassie brilliant blue (CBB) staining. The secreted protein preparations from LGG-WT and LGG-MTs differed in the level of a protein (designated protein X) that migrated as a polypeptide of approximately 40 kDa (Fig. 1A). Specifically, protein X levels in LGG-MT_K56N_ and LGG-MT_K56T_ were significantly increased compared to those in LGG-WT (Fig. 1B), while the corresponding band could not be detected in the secreted-protein preparation obtained from LGG-MT_R99C_. Peptide mass fingerprinting (PMF) analysis by matrix-assisted laser desorption ionization–time of flight mass spectrometry (MALDI-TOF MS), which was performed to identify secreted proteins in bands that migrated at approximately 40 kDa, revealed that protein X was in fact glyceraldehyde-3-phosphate dehydrogenase (GAPDH).

### Characterization of LGG-MTs: morphological observations and growth rates.

Morphological observations of LGG-WT and LGG-MT_K56N_ were performed by scanning electron microscopy (SEM) (Fig. 1C). Cells of LGG-MT_K56N_ were found to have a smaller diameter than those of LGG-WT and were curvilinear, in contrast to the linear shape of LGG-WT (Fig. 1C). Growth curves indicated that LGG-WT, LGG-MT_K56R_, and LGG-MT_R99C_ had comparable growth rates, while the other LGG-MTs had reduced growth rates compared to LGG-WT (data not shown). The largest effect appeared to be a decreased growth rate in LGG-MT_K56N_ compared to the parent strain (Fig. 1D).

### Detection of GAPDH.

**FIG 2:**
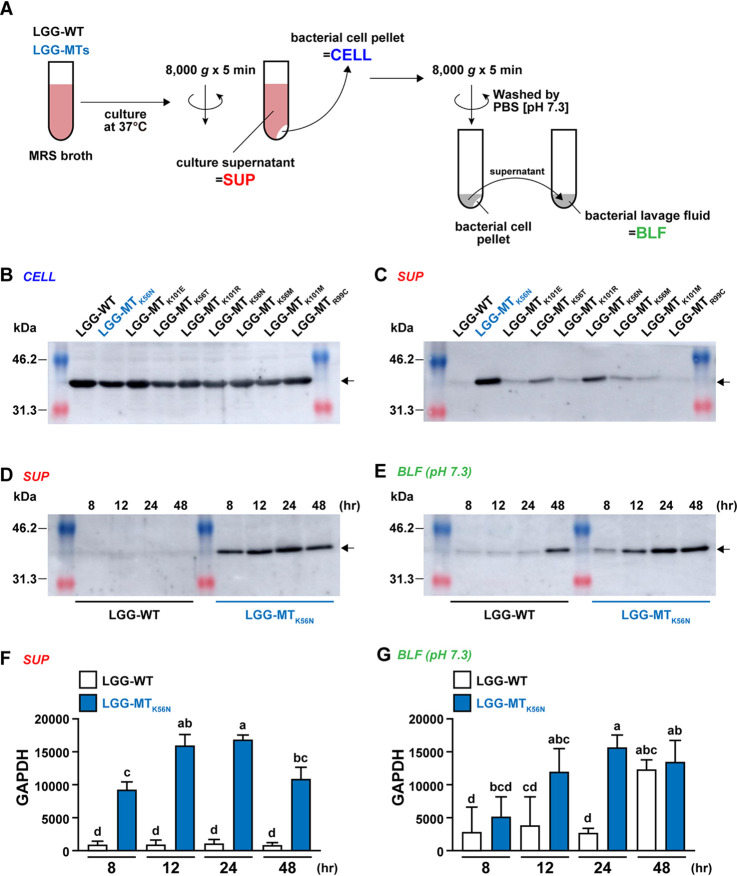
(A) Scheme of the sample preparation procedure. (B and C) GAPDH production by LGG-WT and LGG-MT_K56N_ was determined by Western blot analysis of bacterial cell lysates (B) or supernatants (spent culture medium) (C). (D and E) Time-dependent display of GAPDH in LGG-WT and LGG-MT_K56N_ was monitored at 8, 12, 24, and 48 h in supernatants (spent culture medium) (D) and in BLF (obtained by rinsing the bacterial cell pellet with PBS at pH 7.3) (E). Arrows indicate GAPDH (B, C, D, and E). (F and G) Densitometric analysis of Western blots from three independent experiments was used to quantity GAPDH. Letters (i.e., a, b, and c) represent significant differences (*P* < 0.05) by two-tailed one-way ANOVA. Data are means and SD (*n* = 3). CELL, bacterial cells; SUP, culture supernatant; BLF, bacterial lavage fluid.

Western blotting using an anti-GAPDH antibody was performed to confirm the results of the PMF analysis. Preparations of secreted cellular proteins and bacterial lavage fluid (BLF) were obtained following bacterial growth in MRS broth for 24 h (Fig. 2A). No differences in the intensities of the GAPDH bands were seen between LGG-WT and LGG-MTs in the cellular protein preparations (Fig. 2B). In contrast, the GAPDH band intensities were significantly elevated in the culture supernatants (SUPs) of LGG-MT_K56N_, LGG-MT_K56T_, and LGG-MT_K56M_ compared to LGG-WT (Fig. 2C). To address the time course of GAPDH expression, cultures of LGG-WT and a representative mutant (LGG-MT_K56N_) were incubated at 37°C; proteins from the SUP and BLF (obtained by washing the cell pellet with phosphate-buffered saline [PBS], pH 7.3) were prepared at 8, 12, 24, and 48 h (Fig. 2D and E). The GAPDH band intensities in the SUP and BLF of LGG-MT_K56N_ were nominally greater than those of LGG-WT (Fig. 2F and G), with significant differences observed for SUP at 12, 24, and 48 h (Fig. 2F).

### GAPDH displayed on the cell surface.

**FIG 3:**
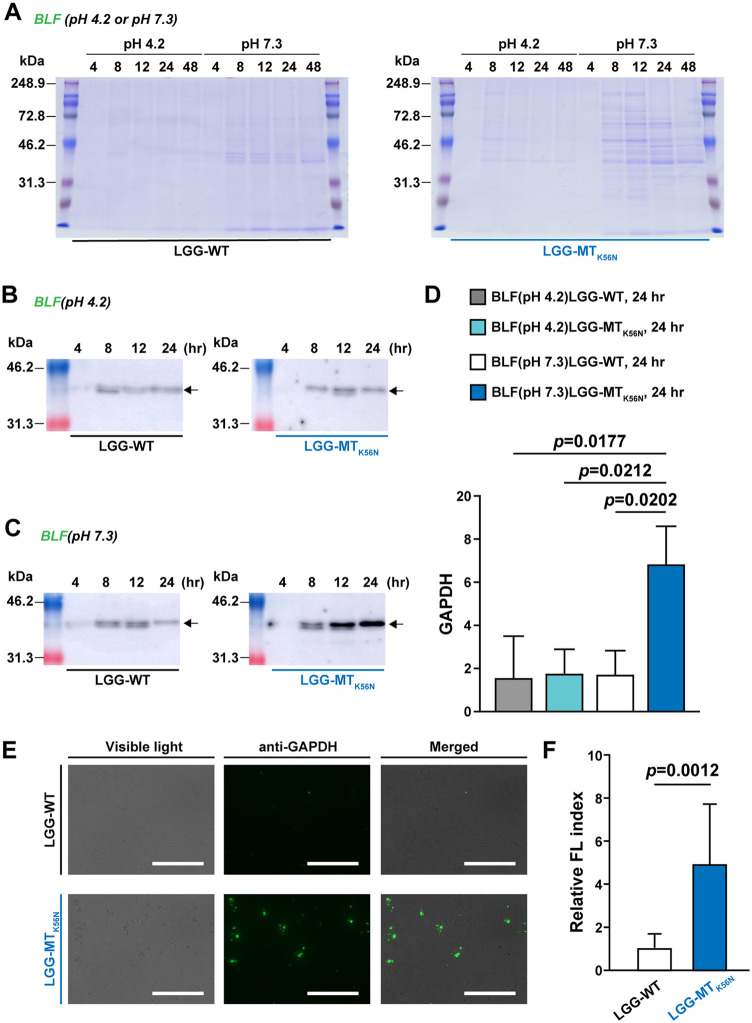
BLFs obtained by rinsing of cell pellets with PBS (at pH 4.2 or 7.3) were analyzed by SDS-PAGE (A), GAPDH Western blotting (B and C), and an EVOS FL imaging system (E). Arrows indicate GAPDH (B and C). Densitometric analysis of Western blots from three independent experiments was used to quantify GAPDH (D). Alexa Fluor 488-GAPDH-stained LGG was analyzed under visible light and fluorescence and as a merged image with an FL imaging system (E). Bar = 100 μm. Fluorescence index values are displayed relative to those obtained from LGG-WT (F). Statistical analysis was performed by a Student's *t* test. Data are means and SD (*n* = 3). BLF, bacterial lavage fluid.

Next, we repeated the time course analysis of SUPs and BLFs from LGG-WT and LGG-MT_K56N_. BLF was generated by rinsing with PBS at either of two pHs (4.2 or 7.3) to characterize GAPDH binding to the LGG cell surface (Fig. 3A to D). For both LGG-WT and LGG-MT_K56N_ of BLF, various sizes of proteins were observed by CBB staining (Fig. 3A). The difference between LGG-WT and LGG-MT_K56N_ was particularly apparent for BLF obtained by rinsing with PBS at pH 7.3. In contrast, extracellular proteins that were eluted by rinsing with PBS at pH 7.3 appeared to be retained by the cells during rinsing with PBS at pH 4.2 (Fig. 3B and C). Notably, the GAPDH levels in the 24-h BLF (pH 7.3) from LGG-MT_K56N_ were significantly elevated compared to those from LGG-WT and those in the 24-h BLF (pH 4.3) from LGG-WT and LGG-MT_K56N_ (Fig. 3D). Bacterial surface display of GAPDH was also observed by fluorescence microscopy (Fig. 3E). For this purpose, bacterial pellets were washed with PBS at pH 4.2 to detect extracellular proteins. The cell surface area labeled with fluorescent antibody was significantly greater in LGG-MT_K56N_ than LGG-WT, indicating that the GAPDH level was increased on the cell surface of the mutant compared to that of the parent (Fig. 3F).

### Adherence to human colonic mucin.

**FIG 4:**
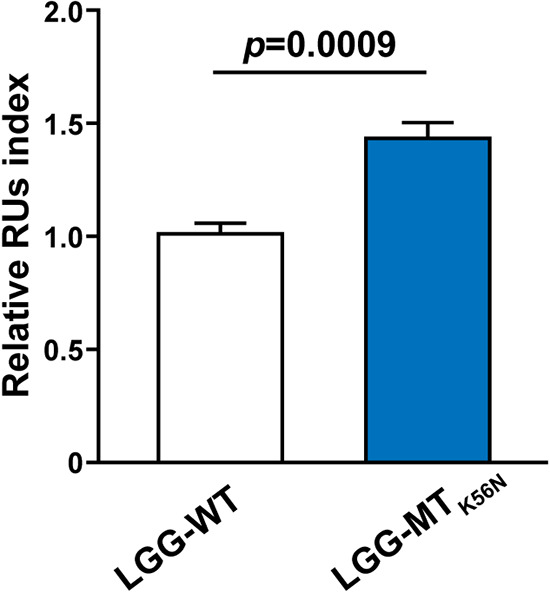
Test of binding of LGG-WT and LGG-MT_K56N_ to human colonic mucin using Biacore analysis. The adhesion values are expressed as RUs (resonance units) at 200 s after cessation of sample addition. Data are expressed as means and SD (*n* = 3).

Next, we conducted experiments using Biacore to verify whether an increased display of GAPDH on the bacterial surface affects adherence to human colonic mucin. Biacore is a monitoring system that measures interactions between biomolecules in real time by biophysical interaction analyses (BIAs). Lyophilisates of LGG-WT and LGG-MT_K56N_ were suspended in HBS-EP buffer (GE Healthcare) to total lyophilized bacteria concentrations of 1.0 mg/ml. Purified type A human colonic mucin (immobilized on a CM5 sensor chip via amino-coupling reactions) was subjected to analysis using a Biacore 1000 system, and resonance unit (RU) values were determined. Significantly higher RU values were observed with LGG-MT_K56N_ than LGG-WT (Fig. 4).

## DISCUSSION

RE can effectively enhance microbial potential by using antibiotics to induce spontaneous mutations in the ribosome and/or RNA polymerase (17). Streptomycin, an aminoglycoside antibiotic often used in RE, binds to the ribosomal 30S subunit and inhibits the initiation of protein synthesis (19). Bacterial mutants resistant to high concentrations of streptomycin have been reported to harbor mutations in the *rpsL* gene, which encodes the 30S ribosomal protein S12 (18). LGG-MTs with *rpsL* mutations were obtained by selecting for spontaneous mutants able to grow with streptomycin at the MIC. Interestingly, the protein preparations from culture supernatants revealed that LGG-WT and the LGG-MTs differed in the density of a band at approximately 40 kDa, which accumulated to significantly higher levels in LGG-MT_K56N_ and LGG-MT_K56T_ than in LGG-WT. In a previous study of LGG-secreted soluble proteins, Shen et al. reported that oral administration of p40 molecules purified from the LGG supernatant to neonatal mice resulted in decreased colitis in adulthood (20). When screening other proteins, Gao et al. detected intestinal barrier-protective effects associated with a novel protein (HM0539) present in the culture supernatant of LGG (21). In addition, Sánchez et al. observed that LGG secreted phosphoglycerate kinase, GAPDH, and transcriptional regulators (22). In the present study, we performed PMF analysis and Western blotting to identify GAPDH as the approximately 40-kDa molecule enriched in the spent medium of the LGG-MTs.

Although GAPDH serves primarily as a metabolic enzyme in cells, it is also a multifunctional “moonlighting protein” (23) that is capable of effecting the transport of tRNA out of the nucleus (24, 25) and shows transfer-regulating activity (26). Notably, in LAB, GAPDH has also been shown to serve as an adhesion factor for human colonic mucin (27). A test to verify the attachment to type A, B, and H blood group antigens showed that GAPDH displayed on the cell surface recognized and bound to the conformation of mucin blood group antigens, exhibiting adhesion (28). Thus, we investigated the amount of GAPDH on the cell surface, hypothesizing that the increased expression of GAPDH led to the enhanced adhesion to mucin. GAPDH is known to be negatively charged in a LAB medium under acidic conditions and is electrostatically bound to the cell wall. Washing cells with a buffer solution above the isoelectric point (IEP) dissociates this bond and releases GAPDH (29). Indeed, work with *Lactiplantibacillus plantarum* LA318 demonstrated that PBS-washed cells showed reduced adherence to human colonic mucin (27). The IEP of GAPDH is 5.3 in *L. plantarum* (29) and 5.53 in LGG (as calculated from the protein structure [data not shown]). Therefore, in both strains, GAPDH is expected to be negatively charged when washed with buffer at pH 7.3, which would cause the protein to be liberated from the outer surface of the cell.

We detected a large amount of GAPDH in the BLF of LGG-MT_K56N_ washed with PBS (pH 7.3) compared with LGG-WT. This GAPDH level was decreased when the BLF was washed with PBS at pH 4.2. Furthermore, imaging following staining with fluorescently labeled anti-GAPDH antibody revealed that GAPDH remained associated with the cell surface following washing with buffer at pH 4.2 but was depleted following washing with buffer at pH 7.3, which corresponds to the data obtained by Saad et al. for *L. plantarum* 299v (30). These results indicated that LGG-MT_K56N_ expressed a large amount of GAPDH on the cell surface compared with LGG-WT. It is reported that ATP-binding cassette (ABC) transporters are involved in the multidrug resistance of LAB (31). In addition, Kinoshita et al. reported that GAPDH produced in *L. plantarum* LA318 may be transported out of the cell via ABC transporters (27). Therefore, ABC transporter expression is considered to affect the increase in GAPDH on the cell surface of LGG-MT_K56N_. In fact, the functional inhibition of ABC transporters decreased the GAPDH level in the SUP and BLF of LGG-MT_K56N_ (Fig. S1).

Next, we assessed whether the enhanced display of GAPDH on the cell surface influenced the adhesion to human colonic mucin. In this study, we chose to employ the Biacore system, which has the advantage of high reproducibility, given that the procedure (from the addition of the bacteria through cleaning) is carried out consistently in all machines (32). Specifically, we evaluated adherence to purified type A human colonic mucin by LGG-WT and LGG-MT_K56N_. Results of the Biacore assay indicated that LGG-MT_K56N_ enhanced adhesion to purified human type A colonic mucin compared with the parent, which strongly indicates that LGG-MT_K56N_ improved the binding ability to the human intestinal tract. In contrast, Sánchez et al. reported that GAPDH secreted by LGG did not bind with mucin and fibronectin (22). However, these results are controversial, given that the origin of the mucin is unknown, the SDS-PAGE sensitivity is very low, and the adherence of bacterial cells was not examined. In the present study, RE altered the BLF protein profile as well as GAPDH production and cell morphology. Further research is necessary to identify other factors that affect the adhesive ability of LGG-MT_K56N_.

In conclusion, we applied RE to the LAB LGG and found that the resulting mutant LGG-MT_K56N_ showed enhanced adhesion to human colonic mucin. The frequency of obtaining high-GAPDH-expression mutants of LGG using streptomycin was 46.0%. The accumulation of GAPDH on the cell surface was suggested to be involved in the improved adhesiveness. Considering the randomness of mutations and ethical issues, there are obstacles to applying conventional microbial breeding technologies such as UV irradiation, chemical mutagenesis, and gene editing and engineering to probiotics. In contrast, RE has a great advantage as a breeding strategy for food-grade probiotics, in that it induces spontaneous mutations mainly in *rpsL* gene. However, given that it is not enough to validate the impact of RE on LGG by evaluating the protein expression, a more detailed impact assessment is needed. We anticipate that this technology will contribute to improving the QOL of humans around the world.

## MATERIALS AND METHODS

### Ribosome engineering of LGG.

*Lacticaseibacillus rhamnosus* GG (LGG; ATCC 53103; ATCC, Manassas, VA, USA) was the wild-type parent (also referred to as LGG-WT) for this experiment. Cultures were grown in de Man, Rogosa, and Sharpe (MRS) broth or agar, supplemented with streptomycin where indicated. For the RE selection, LGG was grown in MRS as a static broth culture overnight at 37°C. The resulting culture was adjusted (using fresh MRS) to 2 × 10^8^ CFU per ml; an aliquot (500 μl, 1 × 10^8^ CFU) was then plated to MRS broth containing streptomycin at 0, 2, 4, 8, 16, 32, 64, 128, 256, 512, and 1,024 μg/ml. The resulting plates were grown as static cultures at 37°C for 3 days. All individual colonies from the 256-μg/ml plate were assigned an identification number, cultured on MRS agar containing 0 μg/ml streptomycin, and incubated at 37°C for 3 days. Isolates that were subsequently cultured on MRS agar containing 256 μg/ml streptomycin and confirmed to grow were considered streptomycin resistant (LGG-MT). The *rpsL* gene was amplified from each isolate and subjected to DNA sequencing using originally designed primers: sense primer, 5′-GGC TGA CGC ATA TTC TGT CTA TAC CG-3′; antisense primer, 5′-GTT GTC CGG ACG TGC TGA CT-3′. Sequencing analysis showed that the amplified products were identical to the *rpsL* gene, and the presence of 8 distinct missense mutations was observed.

### Growth curve and streptomycin sensitivity.

Growth of LGG-WT and LGG-MTs was monitored by optical density measurement at a wavelength of 660 nm (OD_660_) using a spectrophotometer. The OD_660_ was determined at 0, 12, 24, and 48 h for cultures grown in MRS broth without streptomycin. To assess streptomycin sensitivity, bacteria were cultured in MRS broth supplemented with streptomycin at 64, 128, 256, 512, 1,024, 2,048, 4,096, 8,192, 16,384, 32,768, and 65,536 μg/ml; after 16 h of incubation at 37°C, growth was assessed by visual inspection of turbidity. The MIC was defined as the lowest streptomycin concentration that prevented growth under these conditions.

### Analysis of secreted proteins.

Cultures of LGG-WT and the eight LGG-MTs were inoculated at 1:20 into MRS broth and then incubated as static cultures at 37°C for 24 h. Each culture then was subjected to centrifugation (8,000 × *g*, 4°C, 5 min) to separate the pellet (cells) from the culture supernatant (spent medium). An aliquot (1,500 μl) of the spent medium was combined with 300 μl of 100% (wt/vol) trichloroacetic acid (TCA; Wako Pure Chemical Industries, Osaka, Japan); the mixture was vortexed and then placed on ice for 3 h. The precipitate was collected by centrifugation (20,000 × *g*, 4°C, 15 min), and the resulting pellet was subjected to two rounds of washing with 400 μl of acetone followed by centrifugation (20,000 × *g*, 4°C, 15 min) to extract any remaining TCA. The washed pellet was dried in a heating block at 55°C and then dissolved in 50 μl of 0.05 M NaOH. The resulting suspensions were subjected to SDS-PAGE followed by Coomassie brilliant blue (CBB) staining. Bands of interest were excised and submitted for peptide mass fingerprinting (PMF) analysis (Cosmo Bio Co., Ltd., Tokyo, Japan) by MALDI-TOF MS to identify the corresponding proteins.

### Western blotting for GAPDH.

Bacterial cells were suspended in 400 μl of Tris-buffered saline (TBS) with a protease inhibitor cocktail (Roche, Mannheim, Germany); the resulting suspension was transferred to a 2-ml screw-cap microcentrifuge tube containing 0.4 g of 0.2-mm glass beads. Tubes were subjected to 5 rounds of shaking with a bead crusher at 3,200 rpm for 1 min alternating with 1-min incubations on ice. To assess cell surface display of GAPDH, bacterial cells were centrifuged (8,000 × *g*, 4°C, 5 min) and the supernatant was decanted. The pellet then was subjected to 3 rounds of washing with PBS at pH 4.2 or pH 7.3, followed by centrifugation (20,000 × *g*, 4°C, 15 min). Following the final centrifugation, the pellets (corresponding to cell debris and insoluble material) and supernatants (corresponding to soluble released material) were analyzed separately by Western blotting using a primary antibody (polyclonal rabbit anti-GAPDH antibody, 1/5,000; GeneTex, CA, USA) and a secondary antibody (anti-rabbit IgG [whole molecule] peroxidase antibody, 1/5,000; Sigma-Aldrich, MO, USA). Labeling was detected using ImageQuant LAS 500 (GE Healthcare, WI, USA).

### Fluorescent imaging of cell surface GAPDH.

LGG-WT and LGG-MT_K56N_ were inoculated at 1:20 into MRS broth and then incubated as static cultures at 37°C for 24 h. After centrifugation (8,000 × *g*, 4°C, 2 min) and decanting of the supernatant, the bacterial cell pellets were subjected to 3 rounds of washing with PBS at pH 4.2 or 7.3. The resulting pellets were incubated with the primary antibody (polyclonal rabbit anti-GAPDH antibody, 1/1,000; GeneTex) at 4°C for 1 h. The samples then were stained with a secondary antibody (goat anti-rabbit IgG heavy plus light chain [H+L; Alexa Fluor 488], 1/1,000; Abcam, MA, USA) at 4°C for 1 h. Stained bacteria were observed with an EVOS FL Auto imaging system (Thermo Fisher Scientific, MA, USA). The fluorescent area was measured using ImageJ software (NIH, MD, USA).

### Imaging by scanning electron microscopy.

Cultures of LGG-WT and LGG-MT_K56N_ were washed with PBS and then resuspended in fresh PBS. An aliquot (35 μl) of the resulting suspension was transferred to a Nanopercolator filter (JEOL, Tokyo, Japan). The liquid was removed using a 10-ml syringe, and the cells were fixed by immersion of the filter in 2.5% glutaraldehyde (TAAB Laboratories Equipment, Ltd., Berks, United Kingdom) with 2.0% paraformaldehyde prepared in 0.1 M cacodylate buffer for 1 h at room temperature. Following fixation, the plasma membrane was stained by 3 gentle washes with 0.1 M cacodylate buffer and soaking for 1 h in 1% osmium tetroxide. After dehydration by passage through a series of ethanol solutions, each filter was subjected to 2 rounds of incubation (52°C, 30 min) in *t*-butyl alcohol and then stored at −80°C until analyzed. After overnight lyophilization using a DC401 freeze-dryer (Yamato Scientific, Tokyo, Japan), osmium coating was performed using a Neoc-AN osmium coater (Meiwafosis Co., Ltd., Tokyo, Japan). SEM images were collected using a JSM-7600F SEM (JEOL) at an accelerating voltage of 5 kV.

### Assay of adherence to human colonic mucin using Biacore.

The Biacore assay was performed as described previously (32). Freeze-dried bacterial samples were suspended in HBS-EP buffer (GE Healthcare) to a concentration of 1.0 mg/ml. In the present study, purified type A human colonic mucin (28) was immobilized on a CM5 sensor chip (GE Healthcare). The assay was repeated three times.

### Statistical analysis.

All statistical analyses were performed using Prism software (v.7; GraphPad, San Diego, CA, USA). *P* values were calculated using an unpaired Student's *t* test for validation of the Biacore assay or using one-way analysis of variance (ANOVA) followed by the Tukey-Kramer test to detect statistically significant differences. All comparisons were performed as two-tailed tests; *P* values of <0.05 were considered significant. Results are expressed as means and standard deviations (SD).

## Supplementary Material

Supplemental file 1
