# Supplementary material for: Ribosome-Engineered Lacticaseibacillus rhamnosus Strain GG Exhibits Cell Surface Glyceraldehyde-3-Phosphate Dehydrogenase Accumulation and Enhanced Adhesion to Human Colonic Mucin
Source: Appl Environ Microbiol. 2020 Oct 1;86(20):e01448-20. doi: 10.1128/AEM.01448-20 (PMC7531950; doi:10.1128/AEM.01448-20)
Supplement: Supplemental file 1 [file AEM.01448-20-s0001.pdf]

Supplemental Materials

TABLE S1. Strain profile of LGG-MTs isolated by RE for streptomycin resistance

Colony No.	*rpsL* nu mutation	S12 aa mutation
#001	G→T(168)	K56N
#002	G→T(168)	K56N
#003	G→T(168)	K56N
#005	G→T(168)	K56N
#006	A→G(301)	K101E
#007	G→T(168)	K56N
#008	G→T(168)	K56N
#009	G→C(168)	K56N
#010	A→C(167)	K56T
#011	A→G(301)	K101E
#012	G→T(168)	K56N
#013	G→T(168)	K56N
#014	G→T(168)	K56N
#015	G→T(168)	K56N
#016	G→T(168)	K56N
#017	A→G(301)	K101E
#018	A→G(301)	K101E
#019	G→C(168)	K56N
#020	G→C(168)	K56N
#021	G→T(168)	K56N
#022	A→G(302)	K101R
#023	G→T(168)	K56N
#024	A→G(301)	K101E
#025	G→T(168)	K56N
#026	G→T(168)	K56N
#027	A→T(167)	K56M
#028	A→G(301)	K101E
#030	G→T(168)	K56N
#031	A→G(301)	K101E
#032	G→T(168)	K56N
#033	G→T(168)	K56N
#034	A→G(301)	K101E
#035	A→T(167)	K56M
#037	G→T(168)	K101E
#038	A→G(301)	K101E
#039	G→T(168)	K101E
#040	G→T(168)	K101E
#043	G→T(168)	K101E
#044	A→G(301)	K101E
#045	A→G(167)	K56R
#046	A→G(301)	K101E
#047	A→C(167)	K56T

#048	G→T(168)	K101E
#049	G→T(168)	K101E
#050	G→C(168)	K101E
#051	G→T(168)	K56N
#052	G→C(168)	K56N
#053	A→G(302)	K101R
#055	A→C(167)	K56T
#056	A→C(167)	K56T
#059	A→T(167)	K56M
#060	A→G(301)	K101E
#061	A→G(301)	K101E
#062	G→T(168)	K56N
#063	G→T(168)	K56N
#064	G→T(168)	K56N
#065	A→G(167)	K56R
#066	G→T(168)	K56N
#067	A→G(301)	K101E
#068	G→T(168)	K56N
#069	G→T(168)	K56N
#070	G→C(168)	K56N
#071	A→G(301)	K101E
#072	A→G(301)	K101E
#073	A→G(301)	K101E
#074	G→T(168)	K56N
#075	A→C(167)	K56T
#076	A→G(167)	K56R
#077	G→T(168)	K56N
#078	G→T(168)	K56N
#079	G→T(168)	K56N
#081	G→T(168)	K56N
#082	G→C(168)	K56N
#083	G→T(168)	K56N
#084	A→C(167)	K56T
#085	A→G(301)	K101E
#086	G→T(168)	K56N
#087	G→T(168)	K56N
#088	G→T(168)	K56N
#089	G→T(168)	K56N
#090	A→G(301)	K101E
#091	A→G(301)	K101E
#092	G→T(168)	K56N
#093	A→G(301)	K101E
#094	A→G(301)	K101E
#095	A→T(167)	K56M
#098	G→T(168)	K56N
#099	G→T(168)	K56N
#100	G→C(168)	K56N

#101	G→T(168)	K56N
#103	A→G(301)	K101E
#104	G→T(168)	K56N
#105	A→G(301)	K101E
#106	A→G(302)	K101R
#107	A→G(301)	K101E
#109	G→T(168)	K56N
#110	G→T(168)	K56N
#111	G→T(168)	K56N
#112	A→T(302)	K101M
#113	G→T(168)	K56N
#114	G→T(168)	K56N
#115	G→T(168)	K56N
#116	A→G(167)	K56R
#117	A→G(301)	K101E
#118	G→T(168)	K56N
#119	G→T(168)	K56N
#120	A→C(167)	K56T
#121	G→T(168)	K56N
#122	A→G(167)	K56R
#123	A→G(167)	K56R
#124	A→G(167)	K56R
#125	A→G(167)	K56R
#126	A→G(301)	K101E
#127	A→G(167)	K56R
#128	A→G(167)	K56R
#129	A→G(167)	K56R
#130	A→G(167)	K56R
#131	A→G(167)	K56R
#132	A→G(301)	K101E
#133	A→G(167)	K56R
#134	C→T(295)	R99C
#135	G→T(168)	K56N
#136	-	-
#137	-	-
#138	A→C(310)	-
#139	-	-
#140	-	-
#141	A→T(167)	K56M
#142	-	-
#143	-	-
#144	-	-
#145	A→C(167)	K56T

nu, nucleotide; aa, amino acid

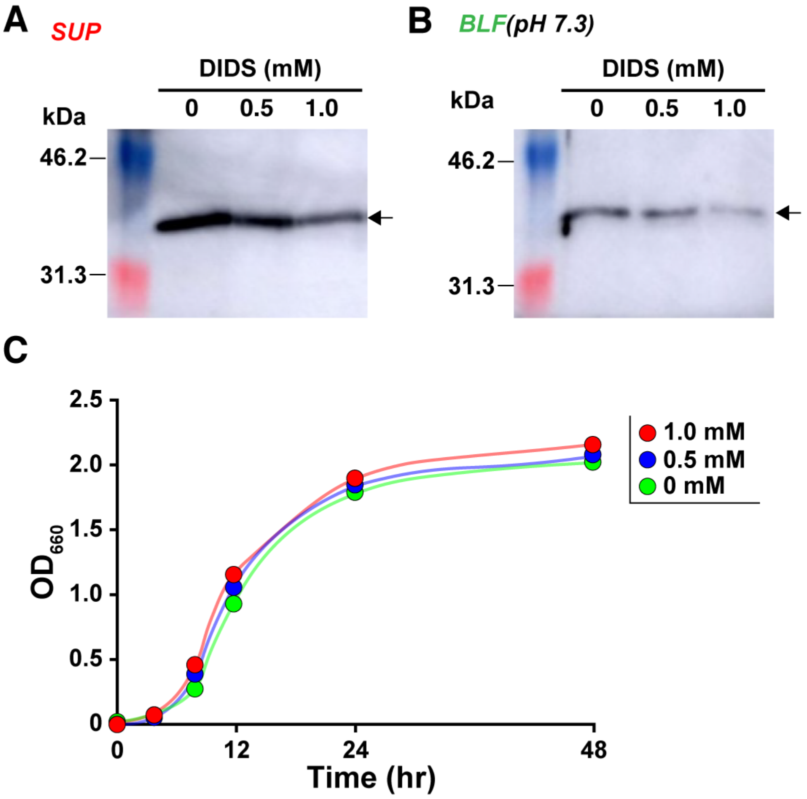

FIG S1. To assess the role of ABC transporters in GAPDH export, we grew cultures for 12 h in MRS broth supplemented with 4,4'-diisothiocyano-2,2'-stilbenedisulfonic acid (DIDS), a compound known to inhibit ABC transporter activity by impeding anion permeation of plasma membrane surfaces (1). LGG-WT and LGG-MT$_{K56N}$ were cultured in MRS broth containing 0, 0.5, and 1.0 mM DIDS, an ABC transporter inhibitor. Western blotting with anti-GAPDH showed that the GAPDH levels were decreased in the SUP (A) and BLF (B) (obtained using PBS at pH 7.3) of LGG-MT$_{K56N}$ grown in the presence of DIDS (compared to levels for cultures grown in the absence of DIDS). Black arrows indicate GAPDH (in A and B). Growth curve (OD_{660}) of LGG-MT$_{K56N}$ cultured in the presence of 0, 0.5, and 1.0 mM DIDS (C). SUP, culture supernatant; BLF, bacterial lavage fluid. These results suggested that LGG exports GAPDH via an ABC transporter. Growth curve analysis confirmed that DIDS did not inhibit cell growth (C).

Reference

1. Hasko G, Deitch EA, Nemeth ZH, Kuhel DG, Szabo C. 2002. Inhibitors of ATP-binding cassette transporters suppress interleukin-12 p40 production and major histocompatibility complex II up-regulation in macrophages. J Pharmacol Exp Ther 301:103-10.
